# Determinants of vaccine acceptance, knowledge, attitude, and prevention practices against COVID‐19 among governmental healthcare workers in Addis Ababa and Adama, Ethiopia: A cross‐sectional study

**DOI:** 10.1002/hsr2.1074

**Published:** 2023-01-18

**Authors:** Aderajew M. Girmay, Mesaye G. Weldegebriel, Melaku G. Serte, Daniel A. Dinssa, Tsigereda A. Alemayehu, Moa A. Kenea, Abel Weldetinsae, Kirubel T. Teklu, Sisay D. Mengesha, Zinabu A. Alemu, Belaynesh Demisie, Bedasa Wagari, Martin R. Evans, Masresha Tessema, Getachew Tollera

**Affiliations:** ^1^ Department of Nutrition and Environmental Health Research Directorate Ethiopian Public Health Institute (EPHI) Addis Ababa Ethiopia; ^2^ Laboratory and Microbiology Consultant, Global Health Programs American Society for Microbiology Huntington New York USA

**Keywords:** control strategies, COVID‐19, healthcare staff, immunization, vaccine

## Abstract

**Background and Aims:**

COVID‐19 vaccines are vital tools for infection prevention and control of the pandemic. However, coronavirus immunization requires acceptance among healthcare workforces and by the community. In Ethiopia, studies focused on determinants of vaccine acceptance, knowledge, attitude, and prevention practices (KAP) contrary to the novel coronavirus among healthcare staff are limited. Hence, closing this gap requires research.

**Methods:**

A cross‐sectional study was conducted on 844 governmental healthcare workers. A stratified, simple random sampling technique was used to select the respondents. Data were collected using a structured questionnaire. Binary and multivariable logistic regression statistical models were used to analyze the data.

**Results:**

This study indicated that only 57.9% of the participants had good COVID‐19 vaccine acceptance, meaning they took at least a dose of the vaccine themselves. We found that 65%, 60.9%, and 51.3% of the participants had good knowledge, prevention practices, and attitude against the pandemic. The novel coronavirus vaccine acceptance rate was 2.19 times more likely among females (adjusted odds ratio [AOR] = 2.19 with 95% confidence interval [CI]: 1.54–3.10) than among male participants. Further, respondents who did not report having any chronic diseases were 9.40 times higher to accept COVID‐19 vaccines (AOR = 9.40 with 95% CI: 4.77, 18.53) than those who reported having a chronic condition. However, healthcare workers who had a habit of chewing khat at least once per week were 4% less likely to take the vaccine (AOR = 0.04 with 95% CI: 0.01, 0.32) than those who had no habit of chewing khat.

**Conclusion:**

Many core factors influencing COVID‐19 vaccine acceptance were identified. A significant number of participants had poor vaccine acceptance, KAP against COVID‐19. Therefore, the government should adopt urgent and effective public health measures, including public campaigns to enhance public trust in COVID‐19 vaccines. In addition, continuous, timely, and practical training should be provided to healthcare workers.

## 1 INTRODUCTION

Coronavirus vaccines are life‐saving therapeutics that prevent or minimize COVID infections and aid in reducing global transmission.^1^, ^2^, ^3^, ^4^ Vaccines provide immunity and prevent or reduce the level of morbidity and mortality in vaccine recipients. Moreover, infections in unvaccinated individuals are minimized once herd immunity is reached.^5^ To achieve this, healthcare workers and other relevant agencies actively participate in prevention and control measures.^6^ However, a successful vaccination campaign requires healthcare workers and community acceptance for successful control and prevention.^7^, ^8^ Because the pandemic infected more than 397 million people and resulted in over 5.7 million deaths.^9^ COVID‐19 vaccination is needed at all health system levels, particularly among healthcare workers.^10^ However, many healthcare workers favored delaying until others have taken the vaccine,^11^, ^12^ perhaps due to fear of side effects and safety and efficacy concerns. Additionally, miss perception and belief about the coronavirus were crucial factors affecting vaccine acceptance.^13^ Furthermore, age, gender, trust in government, training, education, and various socioeconomic variables were identified as determinants of vaccine acceptance.^14^, ^15^, ^16^, ^17^, ^18^ Due to these and other unknown factors, the novel coronavirus vaccine acceptance will be a huge challenge to address, particularly in low‐ and middle‐income countries.^12^

Healthcare workers are critical in providing instruction and factual information to clients, patients, and communities about the threats and benefits of the coronavirus vaccines.^19^ Also, their vaccination helps them to become a role model for patients and communities and increases immunization coverage.^12^ Therefore, knowing the uptake of coronavirus vaccines by healthcare workers is essential for developing appropriate strategies to promote community awareness and uptake of the vaccine.^20^ Despite insufficient research, assessing healthcare workers' knowledge, attitudes, and prevention practices (KAP) against COVID‐19 is essential to reduce the pandemic. These parameters also influence vaccine uptake and the likelihood of recommending vaccines to patients and clients.^21^

While Ethiopia has been tackling the pandemic since March 2020, when COVID‐19 entered the country, many challenges are associated with prevention and control activities. As part of prevention activities, the government started COVID‐19 vaccination in March 2021. The COVID vaccine was offered to all healthcare workers and high‐risk groups. However, little is known about the factors affecting coronavirus vaccine acceptance and the KAP of healthcare workers against the disease. Hence, closing this gap requires research.

## 2 METHODS

### 2.1 Study setting

The Addis Ababa city administration and the Adama city were the study areas. These cities are the most central for visitors and references for patient referrals and are suspected of having a high patient flow in the country. The reason for performing the study in both cities was these assumptions. Addis Ababa is the political and economic center of Ethiopia.^22^, ^23^ It has an estimated total population of 3.65 million people.^24^

Furthermore, according to the unpublished report of 2020 of the *Addis Ababa Food, Medicine, Health Care Administration, and Control Authority*, the city has 1957 governmental and private health institutions. Furthermore, the city has four federal referral hospitals and six regional governmental hospitals. Also, there are 10 subcity health offices and 100 functional governmental healthcare centers. Adama city is located southeast of Addis Ababa at 92 km. The city has six governmental healthcare centers and many private health institutions. In both cities, governmental healthcare centers provide preventive and curative services for existing, emerging, and reemerging infectious and noninfectious diseases. According to a recent report by the *Addis Ababa Health Bureau of 2022*, governmental institutions have an estimated 13,720 healthcare workers. Of these, nearly 5000 are working in governmental healthcare centers. Moreover, according to the current Adama Health Bureau Report, about 300 healthcare workers reside in Adama.

### 2.2 Design and period

A cross‐sectional study was accomplished in Addis Ababa and Adama city, Ethiopia, between July and August 2021.

### 2.3 Source and study population

All governmental healthcare workers found in the study areas were the source population. Additionally, all randomly selected healthcare workers were the study participants. Healthcare workers included anyone who worked in healthcare. These include frontline healthcare workers, such as nurses, midwiferies, physicians, public health officers, pharmacists, laboratory professionals, dentists, and other non‐health professionals, such as finance officers, human resources personnel, cleaners, and guardians. However, workers who had a severe illness and presented on annual or maternity leave were excluded from the investigation.

### 2.4 Sample size and sampling procedure

The required sample size for the study was computed using a single population proportion formula (EPI INFO version 7.2.2.6) with the assumption of a 50% population proportion (since there is no previous study on vaccine acceptance against COVID‐19 in the country), an acceptable margin of error of 0.05 (*e*), and a 95% confidence level (*Z α*/2)

$$\frac{n=Z^{2}\times P\left(1‐P\right)}{e^{2}},$$

$$\frac{n=1.96^{2}\times 0.5\left(1‐0.5\right)=384}{0.05^{2}}.$$

Adding 10% for the nonresponse rate and multiplied by design effect two, the required sample size for this study was 844.

A stratified, simple random sampling method was used to select the study participants. Addis Ababa and Adama city were stratified in the first stage by their administration border. Additionally, listings of the existing healthcare centers were acquired from the Addis Ababa and Adama Health Bureaus. In the second stage, the Addis Ababa healthcare centers were stratified into ten administrative subcities. Besides, Adama, Adama city was stratified into two parts based on the location of healthcare centers. For each subcity and the two parts of Adama city, 30% of healthcare centers were selected using the lottery method.

Moreover, a listing of healthcare workers was obtained from the selected healthcare centers. In the third stage, the healthcare workers were stratified into health professionals (physicians, public health officers, nurses, pharmacists, laboratory professionals, midwives, and dentists) and non‐health‐related personnel (finance officers, human resources personnel, cleaners, and guardians). A proportional sample allocation was conducted based on the number of healthcare workers. The required participants were randomly selected from the listing of healthcare workers. Finally, coordinators provided a self‐directing questionnaire to randomly selected respondents.

### 2.5 Data collection and quality assurance

A structured questionnaire was developed from similar literature.^25^, ^26^ In particular, questions that addressed COVID‐19 prevention practices were taken from similar studies, although modifications were made based on the objective of this study. Two days of training were given to 11 study coordinators. The study coordinators provided a self‐directing questionnaire to the selected respondents at their place of work. Nonresponse was considered if the chosen healthcare worker was not present at a workplace for 2 consecutive working days. To maintain the consistency of the questions, the questionnaire was prepared in English and translated into Amharic and back into English. To enhance data quality, a pretesting was performed on 5% of the total sample size. The tool's internal consistency and validity were examined using Cronbach's *α*, and the value of “*α*” was 0.82. This indicated that the questionnaire was valid and reliable.^27^ In addition, a daily review of a completed questionnaire by the principal investigator and coordinators was done. Questionnaires that were not appropriately administered were rejected.

### 2.6 Study variables

*Dependent variables*: Vaccine acceptance, KAP, and working in an enabling environment were the outcome variables of this study.

*Independent variables*: Sex, age, marital status, religion, education status, type of work, work experience, training, and questions about their health behaviors, including the use of tobacco, alcohol, and khat, were the predictor variables of this study.

### 2.7 Operational definitions

Questions about COVID‐19 vaccine acceptance, KAP, and working in an enabling environment were asked. To determine the vaccine acceptance level against COVID‐19, participants were questioned with one yes or no question about whether they took at least a dose of the COVID‐19 vaccine. Those who received at least a dose of the COVID‐19 vaccine were considered to have good vaccine acceptance, whereas those who did not receive the vaccine were supposed to have poor vaccine acceptance. To assess the presence of an enabling environment for COVID‐19 vaccine uptake, participants were asked 14 yes or no questions. For all items, the presence of an enabling environment was coded as a 1, while the absence was coded as a 0. Those who scored higher or equal to the average value were considered to have a suitable environment for COVID‐19 vaccination, and those who scored below the average value were assumed to be facing a disabling condition to take the COVID‐19 vaccine.

To assess the COVID‐19‐related knowledge of the participants, 17 questions having two choices were prepared. A right response was given a score of 1, while a score of 0 was given for a wrong reply. Total individual responses were summed to calculate the overall knowledge score. Participants who scored above or equal to the average value of the total score were assumed to have good knowledge, and those who scored below the average value were supposed to have poor knowledge. Six questions with two choices were prepared to evaluate the participants' attitudes. A right response was given a score of 1, while a score of 0 was given for a wrong reply. Individual responses were summed for the overall attitude score. Respondents who scored ≥to the average value of the total score were assumed to have a good attitude and those who scored <the average value were considered to have a poor attitude. Seven questions with two choices were prepared to measure the COVID‐19‐related prevention practice of participants. A right response was given a score of 1, while a score of 0 was given for a wrong reply. Individual responses were summed up for the overall preventive practice score. Respondents who scored ≥to the average value of the total score were assumed to have good COVID‐19 prevention practices, and those who scored below the average value were supposed to have poor practices.

The study's ethical clearance was assured in June 2021 by the Ethiopian Public Health Institute with a reference number EPHI 613/138. Written consent was acquired from all participants. The secrecy and privacy of participants were safeguarded all over the investigation process.

### 2.8 Analysis

In the first stage, the data were cleaned and checked for completeness. Since our data were categorical, a Pearson goodness‐of‐fit test was used to check model adequacy,^28^ and we approved that all the expected values were fitted with the observed data. Binary and multivariable logistic regression statistical models were used to assess the association between the coronavirus vaccine acceptance and predictor variables. Having *p* < 0.05 at 95% confidence interval (CI) with a two‐tailed test was statistically significant. The study's results were calculated using Statistical Package for the Social Sciences software version 20.

## 3 FINDINGS

### 3.1 Demographic characteristics of governmental healthcare workers

Overall, 844 healthcare workers were requested to take part in this study. The reply rate was 93.1% for a resulting sample size of 786 respondents. In the study, 63.7% of the respondents were female, and 65.1% were less than 33 years old. The average age of the participants was 33 years. Nearly all (90.1%) healthcare workers were diploma and degree holders. Most (91.6%) of the respondents were healthcare providers, while the remaining 8.4% worked in healthcare centers but did not provide patient care.

Regarding work experience, 61.8% of respondents had less than 8 years of experience. Almost all (91.1%) respondents had no known chronic diseases. Nearly all (97.6%) of the participants did not smoke cigarettes. In addition, 88.7% and 96.9% of the participants did not drink alcohol or chew khat at least once a week, respectively (Table 1).

**Table 1:** Demographic characteristics of governmental healthcare workers (*n* = 786)

Study variables	Frequency	Percent (%)
Sex		
Male	285	36.3
Female	501	63.7
Age group		
<33 years old	512	65.1
≥33 years old	274	34.9
Educational status		
Diploma and degree holder	708	90.1
Masters' degree and above	78	9.9
Type of work		
Healthcare provider	720	91.6
Non‐healthcare related	66	8.4
Work experience, years		
<8	486	61.8
≥8	300	38.2
Marital status		
Single	372	47.3
Married	414	52.7
Residence		
Within the town	667	84.9
Out of the town	119	15.1
One or more known chronic diseases (chronic lung disease, hypertension, diabetes mellitus, heart disease, etc.)		
Yes	70	8.9
No	716	91.1
Cigarette smoker		
Yes	19	2.4
No	767	97.6
Drink alcohol at least once per week		
Yes	89	11.3
No	697	88.7
Chew khat (*Catha edulis*) at least once per week		
Yes	24	3.1
No	762	96.9
Received training about COVID‐19 in the past year		
Yes	177	22.5
No	609	77.5
The average age of the participants	33 years	
Average work experience of the participants	8 years	

### 3.2 Coronavirus vaccine acceptance and enabling environment

In this investigation, the prevalence of receiving at least a dose of the coronavirus vaccine or vaccine acceptance against COVID‐19 was found to be 57.9%. Moreover, 50.4% of the healthcare workers reported being fully vaccinated against COVID‐19. “Fully vaccinated” could be either one or two doses depending upon the manufacturer. Yet, based on the operational definition, only 62.2% of the healthcare workers reported having an enabling or supportive environment to take the vaccine. In the study, 67.8% and 77.5% of the participants reported that the vaccine provision against COVID‐19 was delivered at a convenient time and place, respectively. Moreover, 71% of the respondents said that the benefits and typical undesirable effects of the vaccine against coronavirus were explained in plain terms by different governmental media and 74.2% of the respondents said that the government was making an empowering environment, social inspiration, enhancing motivation, and ensuring adequate vaccine availability. However, only 51.1% of the participants trusted the vaccine against COVID‐19, and only 57.5% of the healthcare workers believed that vaccines against COVID‐19 protected them from becoming seriously ill and dying from COVID‐19 (Table 2).

**Table 2:** Coronavirus vaccine acceptance and enabling environment (*n* = 786)

Study variables	Category	Frequency	Percent (%)
*Vaccine acceptance level against COVID‐19*			
Took at least a dose of the coronavirus vaccine	Yes	455	57.9
	No	331	42.1
Took a full dose of the coronavirus vaccine	Yes	396	50.4
	No	390	49.6
*Enabling the environment to take the coronavirus vaccine*		Answers % (*n*)	
		Yes	No
Was the coronavirus vaccine delivered at a time of day convenient to you?		67.8 (533)	32.2 (253)
Was the coronavirus vaccination given in a close‐by, convenient place to you?		77.5 (609)	22.5 (177)
Were the vaccine's benefits and typical undesirable effects against coronavirus explained in plain terms by different Governmental Media regularly?		71 (558)	29 (228)
Were you building timely faith in the coronavirus vaccine?		51.1 (402)	48.9 (384)
Is the government making an empowering environment, social inspiration, enhancing motivation, and ensuring adequate coronavirus vaccine availability?		74.2 (583)	25.8 (203)
Are coronavirus vaccines harmless for most people 18 years and above?		67.3 (529)	32.7 (257)
Is it also early to know the period of protection for coronavirus vaccines?		89.4 (703)	10.6 (83)
Similar to other vaccines, coronavirus vaccines may not be 100% effective.		72.3 (568)	27.7 (218)
Is the coronavirus vaccination safeguarding you from severe illness and death?		57.5 (452)	42.5 (334)
Although you already have coronavirus, you must be vaccinated when it is provided.		31.6 (248)	68.4 (538)
Ensuring the safety of coronavirus vaccines must be one of the WHO's priorities action?		91.6 (720)	8.4 (66)
Coronavirus vaccines may cause slight side effects, like low‐grade fever, discomfort, or irritation at the injection site.		84.2 (662)	15.8 (124)
Was the government providing timely information about the coronavirus vaccine to healthcare workers?		82.6 (649)	17.4 (137)
Does your religion promote you to take the coronavirus vaccine?		67.8 (533)	32.2 (253)
The average score of the correct answer on enabling environment (%)		70.4 ± 18.9	

Abbreviation: WHO, World Health Organization.

### 3.3 Knowledge of healthcare workers about COVID‐19

In this study, the average score on the knowledge questions was 86.6%. Yet, based on the operational definition, only 65% of the healthcare workers had good knowledge regarding the pandemic. Of the total, 89.6% of the participants knew that the threat of severe infection with coronavirus upsurges with age. Furthermore, 95.2% of the respondents knew coronavirus's most common clinical symptoms, such as dry cough, high fever, and fatigue. Besides, 86.9% of the respondents knew the less common clinical signs of COVID‐19, such as pain, sore throat, headache, diarrhea, loss of taste, and discoloration of fingers.

Moreover, 91% of the respondents knew the severe clinical symptoms of COVID‐19, such as difficulty breathing, chest pain, and loss of speech. In the study, 95.2% of participants knew that regular hand washing with water and soap could eliminate COVID‐19 infection after touching objects. In addition, 96.7% of the participants knew that COVID‐19 is more pathogenic in people with underlying causes, such as cancer, diabetes, and chronic respiratory diseases (Table 3).

**Table 3:** Coronavirus‐related knowledge of the healthcare workers (*n* = 786)

Study variables	Answers % (*n*)	
	Yes	No
Is COVID‐19 a novel strain of coronavirus that has not been formally recognized in persons?	66.7 (524)	33.3 (262)
Is COVID‐19 an infectious disease?	94.9 (746)	5.1 (40)
Is the contributing agent of COVID‐19 a virus?	88.5 (696)	11.5 (90)
Do you know the mean incubation period of the novel coronavirus is 5–6 days and can be as long as 14 days?	94.9 (746)	5.1 (40)
Is the threat of severe disease with coronavirus upsurging with age?	89.6 (704)	10.4 (82)
Are dry cough, high fever, and fatigue the most common clinical signs of COVID‐19?	95.2 (748)	4.8 (38)
Are headaches, sore throat, pain, diarrhea, conjunctivitis, loss of taste, and discoloration of fingers the less common clinical indications of COVID‐19?	86.9 (683)	13.1 (103)
Is the difficulty of breathing, chest pain, and loss of speech grave clinical indicators of the novel coronavirus?	91 (715)	9 (71)
Is the prevalence of COVID‐19 disease increasing at an alarming rate in Ethiopia?	74.4 (585)	25.6 (201)
Can regular hand wash with water and soap after touching objects eliminate the novel coronavirus?	95.2 (748)	4.8 (38)
Is COVID‐19 conveyed directly through cough?	95.2 (748)	4.8 (38)
Is COVID‐19 highly dangerous in people with known chronic diseases?	96.7 (760)	3.3 (26)
Can wearing a facemask help protect you from COVID‐19?	95.9 (754)	4.1 (32)
Does keeping physical distance protect you from COVID‐19?	97.3 (765)	2.7 (21)
Can avoiding crowded places help to prevent COVID‐19?	95 (747)	5 (39)
Is drinking local Araki not preventing COVID‐19?	66.2 (520)	33.8 (266)
Can COVID‐19 be transmitted between humans and animals?	49.2 (387)	50.8 (399)
Percentages mean score of the correct answer to knowledge about COVID‐19	86.6 ± 11.1	

### 3.4 Attitudes of healthcare workers related to the novel coronavirus

In this investigation, the average score of the attitude questions was 72.2%. Yet, depending on the operational definition, only 51.3% of the respondents had a good attitude. Additionally, 90.6% of the healthcare workers thought that continuous health education could help to prevent COVID‐19. Moreover, 81.7% of the healthcare workers believed accurately in the advantage of personal protective device utilization to prevent disease. A similar number (79.6%) reported that eating garlic can boost immunity and may reduce the risk of severity of the coronavirus (Table 4).

**Table 4:** The attitude of healthcare workforces related to the novel coronavirus (*n* = 786)

Study variables	Answers % (*n*)	
	Yes	No
It is my view that continuous health education can help to avert the novel coronavirus.	90.6 (712)	9.4 (74)
It is my view that the available COVID‐19 vaccines should be used.	75.8 (596)	24.2 (190)
I believe that the knowledge related to COVID‐19 in the community is insufficient.	79.6 (626)	20.4 (160)
I believe that eating garlic can boost immunity and may reduce the risk of the severity of the new coronavirus.	79.6 (626)	20.4 (160)
It is my view that the utilization of personal protective devices is effective in preventing COVID‐19.	81.7 (642)	18.3 (144)
I believe the novel coronavirus disease can be transmitted through contact from household pet animals to people.	47.3 (372)	52.7 (414)
Percentages mean score of the correct answer to attitude regarding COVID‐19	72.2 ± 22.0	

### 3.5 Level of the novel coronavirus prevention practices among the healthcare workforce

The average score on the prevention practice questions was 79.4%. Yet, founded on the operational definition, only 60.9% of the healthcare workers used good prevention practices toward COVID‐19. Nearly all participants (95.3%, 91.6%, 97.1%, and 94.3%) maintained personal hygiene, using hand sanitizer, using face masks when out of home, and washing hands regularly to prevent infection (Table 5).

**Table 5:** Prevention practices of healthcare workers regarding COVID‐19 (*n* = 786)

Study variables	Answers % (*n*)	
	Yes	No
To avoid infection and spread of the novel coronavirus, I should prevent communal transports such as buses, taxis, and trains.	43.1 (339)	56.9 (447)
To avert infection and the spreading of the novel coronavirus, I should pay more attention to my hygiene.	95.3 (749)	4.7 (37)
I usually use sanitizer to avoid infection and the spread of the novel coronavirus.	91.6 (720)	8.4 (66)
To avoid infection and to spread the novel coronavirus, I use a facemask usually when out of the home.	97.1 (763)	2.9 (23)
To prevent infection and the spreading of the novel coronavirus, I often wash my hands.	94.3 (741)	5.7 (45)
To avert infection and the spread of the novel coronavirus, I respect the law two meters away when speaking in front of others.	64.2 (505)	35.8 (281)
I evade crowded places to avoid infection and the spread of the novel coronavirus.	70.4 (553)	29.6 (233)
Percentages mean score of the correct answer to prevention practices against COVID‐19	79.4 ± 20.3	

### 3.6 Determinants of the novel coronavirus vaccine acceptance

In the binary logistic regression statistical model, nine explanatory variables, including sex, age, education level, work type, the health status of healthcare workers, not chewing khat at least once per week, receiving training about COVID‐19 in the past year, not drinking alcohol at least once per week, and work experience of healthcare workers, were significantly associated with vaccine acceptance. A multivariable logistic regression analysis was performed to make the rates of vaccine uptake in each study variable the same, minimize confounding factors, and identify real determinant factors of coronavirus vaccine acceptance. In this model, seven determinant factors, including sex, age, educational level, work type of healthcare worker, not reporting a chronic condition, not chewing khat at least once per week, and receiving training about COVID‐19 in the past year, were meaningfully associated (*p* < 0.05) with vaccine uptake (Table 6).

**Table 6:** Multivariable logistic regression analysis (*n* = 786)

Study variables	Vaccine acceptance		*p* Value	AOR with 95% CI
	Yes	No		
Sex of the respondents				
Male	188	97		Reference
Female	267	234	<0.001	2.19 (1.54–3.10)
The age group of the participants				
≥33 years old	180	94		Reference
<33 years old	275	237	0.009	1.74 (1.15–2.64)
Educational status of respondents				
Masters' degree and above	56	22		Reference
Diploma and degree holder	399	309	0.007	2.26 (1.25–4.13)
Healthcare staff's work type				
Non‐health‐related	18	48		Reference
Healthcare provider	437	283	<0.001	3.41 (1.83–6.36)
Have one or more known chronic diseases (chronic lung disease, hypertension, diabetes mellitus, and heart disease)				
Yes	14	56		Reference
No	441	275	<0.001	9.40 (4.77–18.53)
Chew khat (*Catha edulis*) at least once per week				
No	454	308		Reference
Yes	1	23	0.003	0.04 (0.01–0.32)
Received training about COVID‐19 in the past year				
No	339	270		Reference
Yes	116	61	0.007	1.71 (1.16–2.54)

Abbreviations: AOR, adjusted odds ratio; CI, confidence interval.

## 4 DISCUSSION

In Ethiopia, COVID‐19 vaccines have been available to all healthcare workers and high‐risk groups since March 2021. However, the current finding indicates that vaccine uptake of at least a dose remains low. The main reasons might be a lack of clear information, pitfalls, lack of trust, poor understanding of vaccine benefits, or fear of potential side effects of the vaccine. Moreover, vaccine hesitancy could be due to a lack of religious support, as only 67.8% of the participants' religions supported vaccine uptake. Immunization is the most effective medical measure to prevent coronavirus and is considered by the World Health Organization as essential to ending the pandemic.^29^ However, the vaccine hesitancy against COVID‐19 among healthcare workers might be due to being uninformed or misinformed on the safety and efficacy of COVID vaccines.^30^, ^31^ The current finding was lower than a study performed in 2019.^32^ This could be due to the difference in the availability and promotion of COVID educational information or cultural factors. Further, the study's COVID‐19 vaccine acceptance was low compared to other recent studies.^33^, ^34^, ^35^, ^36^, ^37^ A difference in the study period could be the reason for the discrepancy. Also, misinterpretations of information from the prior COVID‐19 pandemic, healthcare workers may be discouraged from taking the COVID‐19 vaccination.

In contrast to studies conducted in other regions of the world, the current study discovered that healthcare workers had a higher level of acceptance for the COVID‐19 vaccination.^38^, ^39^, ^40^, ^41^

Differences in the socio‐demographic characteristics of the healthcare workers and the level of knowledge about the COVID‐19 vaccine may contribute to the observed disparity. Additionally, the differences in the health systems may be a factor for this difference.

In this study, 65% of the health center workers had good knowledge of COVID prevention and control measures, whereas the remaining 35% lacked sufficient knowledge. Compared to an investigation, the knowledge of the healthcare staff was low.^42^ The main reasons for the lack of knowledge among the respondents might be a lack of interest in reading and low interest in following credible sources of scientific knowledge from the media, including the internet. On the other hand, this study showed that healthcare workers had higher knowledge about COVID‐19 than in other studies.^43^, ^44^ This discrepancy may be due to differences in the study time, study region coverage, and topical focus. Although a study has stated there is a probability of spreading COVID‐19 through domestic pets,^45^ the recent study indicated that 50.8% of the participants did not know that COVID‐19 could be transmitted between humans and animals. This revealed a lack of knowledge of COVID‐19 transmission and control methods among the participants.

Moreover, the current study revealed that 51.3% of the participants had a positive attitude against coronavirus. This result was very low compared to a meta‐analysis conducted in 2021 and a study done in Vietnam.^42^, ^46^ This finding indicated that a significant number of healthcare staff had a poor attitude toward coronavirus and calls for strong action. However, this finding was consistent with a study done in 2020.^47^

Furthermore, this study indicated that only 60.9% of the respondents had good prevention practices for COVID‐19. This could be due to the ineffective implementation of available COVID‐19 manuals, guidelines, rules, and regulations. This finding was low compared to a study conducted in 2020.^48^, ^49^ Conversely, the prevention practice against the COVID‐19 among healthcare workers in this study was higher than that reported in Ethiopia.^50^, ^51^ This might be caused by disparities in access to the most recent information, instructions, and manuals. In addition, this study revealed that 33.8% of respondents reported drinking local Araki (colorless distilled alcoholic beverage) can prevent COVID‐19. This could be due to incorrect cultural beliefs and the poor acceptance of scientific ideas.

The findings of this study revealed that coronavirus vaccine acceptance was 2.19 times higher among females (adjusted odds ratio [AOR] = 2.19 with 95% CI: 1.54–3.10) than among male healthcare workers. This indicated that female healthcare workers had a higher sense of responsibility and concern about their health and families than male participants. However, this result needs further research. Furthermore, participants under 33 years old were 1.74 times more likely to accept immunization (AOR = 1.74 with 95% CI; 1.15, 2.64) compared to those whose age group was greater than or equal to 33 years old. This finding was consistent with a study performed in Malaysia and Jordan.^52^ In addition, the likelihood of healthcare workers with educational levels of diploma and degree had 2.26 times greater vaccine acceptance (AOR = 2.26 with 95% CI; 1.25, 4.13) compared to those with higher education levels. This revealed that COVID‐19 vaccine hesitancy was higher among participants with higher education levels. The reason could be a lack of specific awareness about the vaccines, inadequate training, and communication about the safety and efficacy of the coronavirus vaccine, or lower perceived risk among higher educated persons.

Moreover, vaccine acceptance against COVID‐19 was 3.41 times higher among healthcare providers (AOR = 3.41; 95% CI: 1.83‐6.36) than among participants who had non‐health‐related work. As expected, this could be due to the in‐depth knowledge of vaccine benefits and immunity among healthcare providers. Additionally, the study indicated that healthcare workers with no known chronic diseases were 9.4 times higher to receive the vaccine (AOR = 9.4 with 95% CI: 4.77, 18.53) than those with underlying causes. This might be due to fear of side effects, contraindication due to the existing chronic disease(s), or lack of trust in the available vaccines. Healthcare workers who had a habit of chewing khat at least once per week were 4% less likely to take the vaccine (AOR = 0.04 with 95% CI: 0.01, 0.32) than those who had no habit of chewing khat. Healthcare workers who had participated in training during the past year were 1.71 times higher in vaccine acceptance (AOR = 1.71 with 95% CI: 1.16, 2.54) than those who had not received training. This revealed that gaining knowledge through practical training improved vaccine acceptance. The current study indicated that 29% of healthcare workers did not avoid overcrowded places. Despite this finding needs emphasize, it was higher than a study done in 2021.^51^

## 5 CONCLUSION

This study provides insights into vaccine uptake among healthcare workers in Ethiopia, as a significant number of healthcare staff had poor vaccine acceptance, KAP against COVID‐19. Many core determinant factors affecting the coronavirus vaccine acceptance were identified. Vaccine acceptance was higher among female healthcare workers, those less than 33 years old, and those who did not report having a chronic disease. Additionally, the uptake of coronavirus immunization was more likely among trained healthcare staff than those who had not received training. The coronavirus vaccine uptake was less likely among healthcare workers who had a habit of chewing khat at least once per week than those who had no habit of chewing khat. Therefore, the government and other relevant agencies should promote urgent and effective public health measures, including public campaigns to promote and enhance public trust in COVID ‐19 vaccines. Further, continuous, timely, and practical training should be provided to healthcare workers to improve their knowledge, attitude, and infection control and prevention measures against the coronavirus.

### 5.1 Strengths and limitations of the study

The study used a representative sample, providing information on COVID‐19 vaccine acceptance, the KAP of healthcare workers. The study will have a significant impact on creating awareness for the prevention and control of the ongoing COVID‐19 pandemic. However, as the study was cross‐sectional, it only reported associations between predictor variables and vaccine acceptance.

## AUTHOR CONTRIBUTIONS

**Aderajew M. Girmay**: Conceptualization; data curation; formal analysis; funding acquisition; investigation; methodology; project administration; resources; software; supervision; validation; visualization; writing – original draft; writing – review and editing. **Mesaye G. Weldegebriel**: Data curation; formal analysis; funding acquisition; investigation; methodology; project administration; resources; software; supervision; validation; visualization; writing – review and editing. **Melaku G. Serte**: Data curation; investigation; methodology; project administration; resources; software; supervision; validation; visualization; writing – review and editing. **Daniel A. Dinssa**: Data curation; investigation; methodology; project administration; resources; software; supervision; validation; visualization; writing – review and editing. **Tsigereda A. Alemayehu**: Data curation; investigation; methodology; project administration; resources; software; supervision; validation; visualization; writing – review and editing. **Moa A. Kenea**: Data curation; investigation; methodology; project administration; resources; software; supervision; validation; visualization; writing – review and Editing. **Abel Weldetinsae**: Data curation; investigation; methodology; project administration; resources; software; supervision; validation; visualization; writing – review and editing. **Kirubel T. Teklu**: Data curation; investigation; methodology; project administration; resources; software; supervision; validation; visualization; writing – review and editing. **Sisay D. Mengesha**: Data curation; investigation; methodology; project administration; resources; software; supervision; validation; visualization; writing – review and editing. **Zinabu A. Alemu**: Data curation; investigation; methodology; project administration; resources; software; supervision; validation; visualization; writing – review and editing. **Belaynesh Demisie**: Data curation; investigation; methodology; project administration; resources; software; supervision; validation; visualization; writing – review and editing. **Bedasa Wagari**: Data curation; investigation; methodology; project administration; resources; software; supervision; validation; visualization; writing – review and editing. **Martin R. Evans**: Investigation; methodology; project administration; resources; software; validation; visualization; writing – review and editing. **Masresha Tessema**: Investigation; methodology; project administration; resources; software; supervision; validation; visualization; writing – review and editing. **Getachew Tollera**: Investigation; methodology; project administration; resources; software; supervision; validation; visualization; writing – review and editing.

## CONFLICT OF INTEREST

The authors declare no conflict of interest.

## TRANSPARENCY STATEMENT

The lead author Aderajew M. Girmay affirms that this manuscript is an honest, accurate, and transparent account of the study being reported; that no important aspects of the study have been omitted; and that any discrepancies from the study as planned (and, if relevant, registered) have been explained.

## Data Availability

The study's all writers have read and approved the final version of the manuscript. Also, the authors confirm that the data backup of the current study results is accessible within the article and/or its extra resources.
